# Recurrent gastric variceal bleeding due to wandering spleen – complete resolution following laparoscopic splenectomy: A case report

**DOI:** 10.1097/MD.0000000000047197

**Published:** 2026-01-16

**Authors:** Byung Chul Jin, Seung Young Seo, Hyeon Seok Baek, Sang-Wook Kim

**Affiliations:** a Department of Internal Medicine, Division of Gastroenterology and Hepatology, Jeonbuk National University Medical School, Research Institute of Clinical Medicine of Jeonbuk National University-Biomedical Research Institute of Jeonbuk National University Hospital, Jeonju, South Korea.

**Keywords:** gastric varices, laparoscopic splenectomy, sinistral portal hypertension, splenic vein torsion, wandering spleen

## Abstract

**Background:**

Wandering spleen is a rare clinical entity caused by laxity or absence of splenic suspensory ligaments, predisposing to torsion of the vascular pedicle and subsequent vascular compromise. In unusual cases, splenic vein torsion may lead to left-sided (sinistral) portal hypertension with isolated gastric varices in the absence of cirrhosis. Although uncommon, variceal bleeding from this mechanism can be severe and requires timely recognition.

**Case presentation:**

We report a 56-year-old man who presented with recurrent melena. Esophagogastroduodenoscopy revealed large fundic gastric varices without esophageal varices, and cross-sectional imaging identified a wandering spleen displaced into the pelvic cavity with venous congestion but no evidence of liver disease. Endoscopic cyanoacrylate injection provided temporary hemostasis; however, because of persistent vascular compromise and risk of rebleeding, laparoscopic splenectomy was performed. The postoperative course was uneventful, and follow-up computed tomography 5 months later confirmed complete resolution of perigastric varices and splenic venous congestion, demonstrating the reversibility of sinistral portal hypertension once splenic outflow obstruction is corrected.

**Conclusion:**

Wandering spleen with splenic vein torsion should be considered in patients presenting with isolated gastric varices and no signs of liver cirrhosis. While endoscopic therapy may achieve transient hemostasis, it does not correct the underlying hemodynamic disturbance. Splenectomy represents the definitive treatment, achieving durable hemodynamic correction and preventing further bleeding. Early recognition and surgical management are therefore essential for optimal outcomes in this rare but clinically significant condition.

## 1. Introduction

Wandering spleen is a rare condition caused by congenital or acquired laxity of the splenic suspensory ligaments, which results in increased mobility of the spleen within the abdominal cavity.^[1]^ The incidence of splenic abnormalities is estimated to be <0.2 %, with most cases occurring in women of reproductive age.^[2]^ The resulting hypermobility predisposes the spleen to torsion of the splenic vascular pedicle, which can lead to complications such as splenic congestion, infarction, hypersplenism, and, in rare cases, vascular obstruction.^[3–5]^

A less common but clinically significant consequence of splenic torsion is splenic vein obstruction, which can result in increased pressure in the short gastric veins and development of isolated gastric varices (IGV).^[6]^ This condition, known as left-sided (sinistral) portal hypertension, occurs in the absence of liver disease or generalized portal hypertension and may lead to life-threatening upper gastrointestinal bleeding.^[3]^

Although a few case reports have described gastric variceal bleeding associated with wandering spleen, this condition remains poorly recognized in clinical practice.^[7]^ Prompt diagnosis using cross-sectional imaging and endoscopy is essential. When splenic vascular compromise is identified, laparoscopic splenectomy is considered the definitive treatment to resolve the underlying cause of the varices and prevent recurrence.^[8]^

We report the case of a 56-year-old male who presented with recurrent upper gastrointestinal bleeding due to IGV associated with a torsed, wandering spleen. Surgical removal of the spleen led to the complete resolution of the varices on follow-up imaging. This case highlights the importance of including wandering spleen in the differential diagnosis of IGV in patients without cirrhosis. This case report was approved by the Institutional Review Board of Jeonbuk National University Hospital (IRB No. CUH 2025-05-066).

## 2. Case presentation

**Figure 1.**
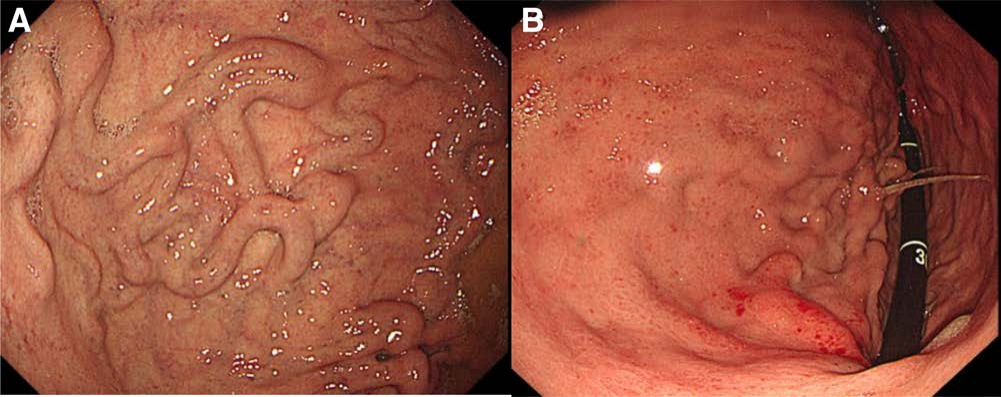
Endoscopic findings of gastric varices. (A) Surveillance EGD performed at our hospital in November 2022 revealed IGV (type I) in the gastric fundus without evidence of bleeding. (B) EGD performed on admission (December 13, 2023) demonstrated large, tortuous fundic varices without evidence of bleeding. IGV = isolated gastric varices, EGD = esophagogastroduodenoscopy.

A 56-year-old man presented to the emergency department with a 2-day history of melena. He denied having hematemesis, abdominal pain, or the use of nonsteroidal anti-inflammatory drugs. He had a history of IGV, which were first detected incidentally during a routine health check esophagogastroduodenoscopy (EGD) at another hospital in 2017, demonstrating IGV type I without bleeding stigmata. As the patient had no symptoms or evidence of portal hypertension at that time, he was managed conservatively and remained stable without gastrointestinal bleeding until the current episode. A follow-up EGD performed at our hospital in November 2022 again revealed IGV at the gastric fundus (Fig. 1A). The patient had no known liver disease or a history of endoscopic therapy. His past medical history included an inguinal hernia repair in 2023. He reported habitual alcohol consumption of approximately 2 pints of vodka 5 to 6 days per week, without a history of smoking. The patient was hemodynamically stable on admission. Physical examination was unremarkable except for melena, which was confirmed on performing a digital rectal examination. Laboratory tests revealed a hemoglobin level of 9.5 g/dL, with normal liver enzyme levels, platelet counts, and coagulation profile. The initial EGD on admission (December 13, 2023) revealed large fundic varices (IGV type I) without stigmata of recent bleeding (Fig. 1B).

**Figure 2.**
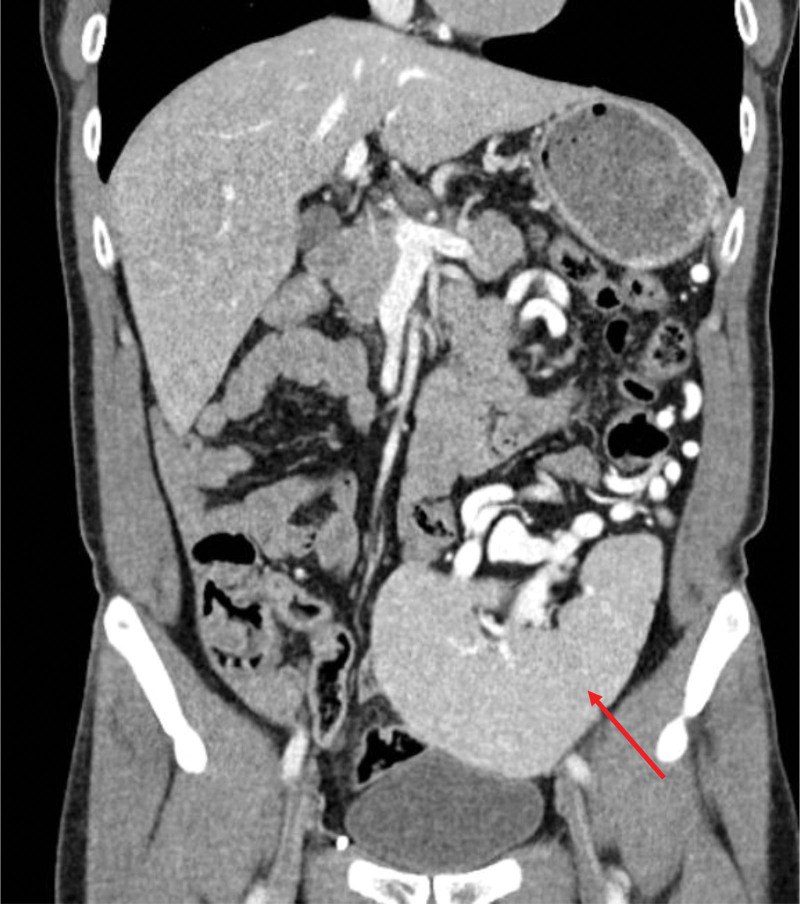
Initial abdominal CT performed on December 12, 2023 (hospital day 1) showing a large volume of hypodense material filling the stomach lumen, suggesting recent upper gastrointestinal bleeding. The spleen is abnormally located in the left pelvic cavity (arrow), consistent with a wandering spleen. Engorged perigastric collateral veins are visualized in the absence of cirrhotic changes. CT = computed tomography.

**Figure 3.**
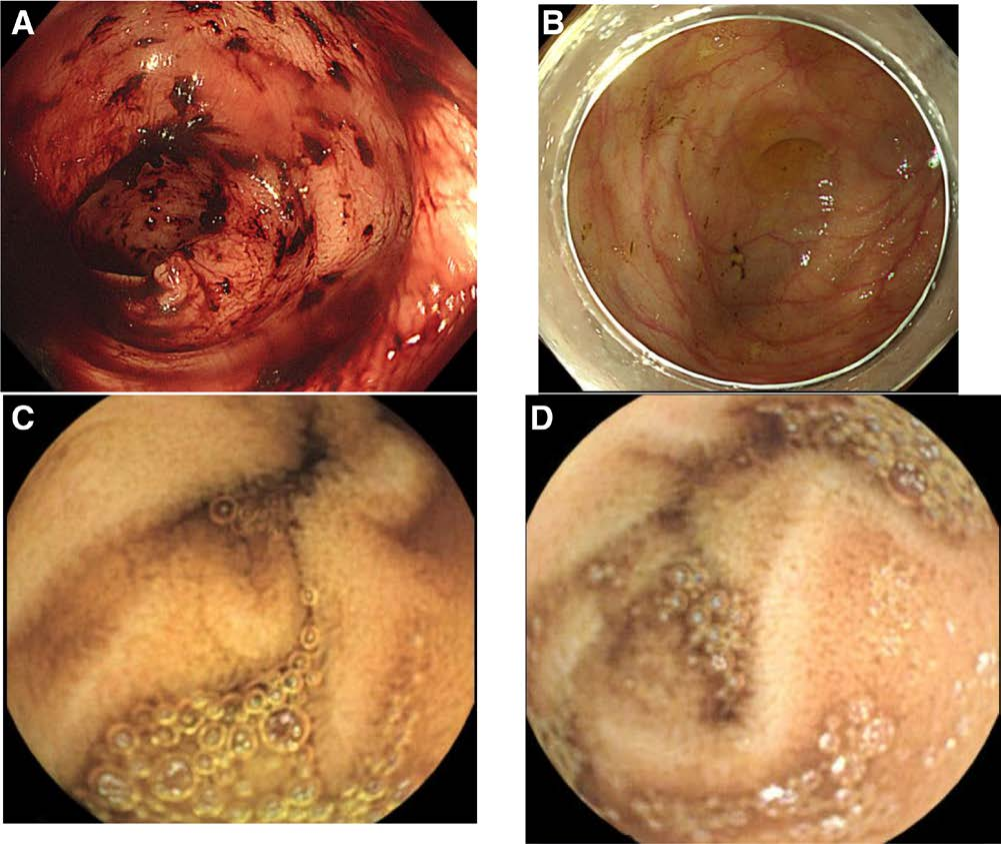
Lower gastrointestinal and capsule endoscopic findings. (A) Sigmoidoscopy on December 13, 2023 (hospital day 1) showing melena in the rectosigmoid colon. (B) Colonoscopy on December 14, 2023 (hospital day 2) demonstrating normal-appearing cecal mucosa. (C) Capsule endoscopy on December 14, 2023 (hospital day 2) showing normal jejunal mucosa without a bleeding focus. (D) Capsule endoscopy image of the distal ileum on the same day showing dark red fluid and yellowish debris, suggestive of prior bleeding without active hemorrhage.

Abdominal computed tomography (CT) performed on December 12, 2023 (hospital day 1) demonstrated a wandering spleen in the left pelvic cavity with splenic vein engorgement and prominent perigastric collateral vessels (Fig. 2). There was no evidence of cirrhosis or portal vein dilation on imaging, despite the patient’s history of significant alcohol consumption. Sigmoidoscopy on December 13, 2023 (hospital day 1) revealed fresh blood in the rectosigmoid colon (Fig. 3A), and colonoscopy on December 14, 2023 (hospital day 2) after bowel preparation showed normal mucosa throughout the colon without signs of active bleeding (Fig. 3B). Capsule endoscopy on December 14, 2023 (hospital day 2) did not identify any specific bleeding focus in the jejunum (Fig. 3C); however, dark red fluid and yellowish material were noted in the distal ileum approximately 4 to 5 hours after ingestion, consistent with old blood (Fig. 3D).

**Figure 4.**
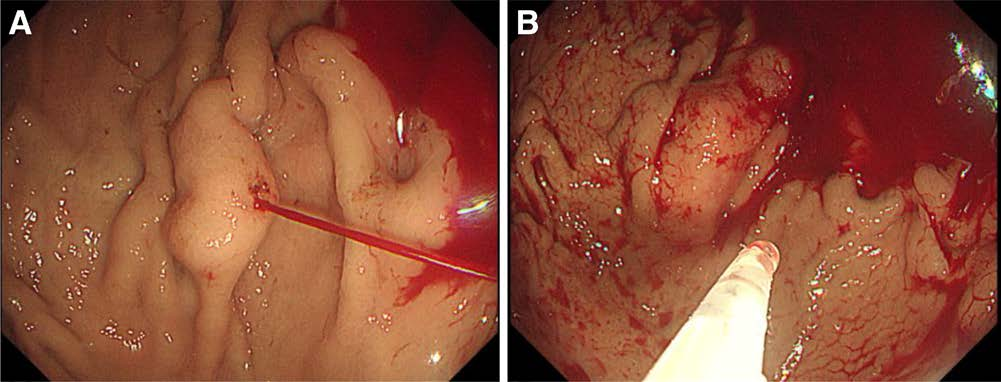
Emergency endoscopy during a rebleeding episode. (A) Active spurting bleeding from a fundic varix observed during EGD on December 20, 2023 (hospital day 9). (B) EVO using cyanoacrylate in progress. EGD = esophagogastroduodenoscopy, EVO = endoscopic variceal obturation.

**Figure 5.**
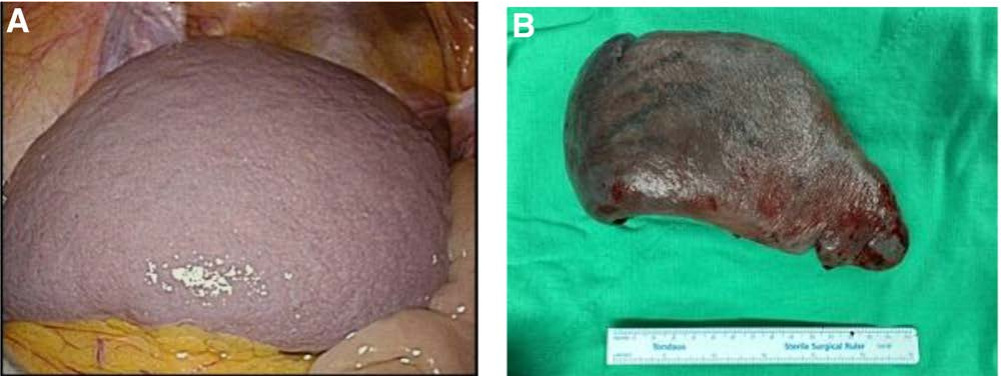
Surgical findings of a wandering spleen. (A) Laparoscopic view during splenectomy on December 28, 2023 (hospital day 17) showing a large, well-encapsulated spleen (~15 cm) located in the pelvic cavity. (B) Gross image of the resected spleen showing an enlarged organ with a congested appearance.

On December 20, 2023 (hospital day 9), the patient developed recurrent melena. Repeat EGD revealed an active spurting hemorrhage from a fundic varix, which was successfully managed with endoscopic variceal obturation using cyanoacrylate injection (Fig. 4A and B). On December 28, 2023 (hospital day 17), laparoscopic splenectomy was performed given the concern of rebleeding and persistent venous congestion. Intraoperatively, the spleen was enlarged and located in the pelvic cavity surrounded by engorged varices (Fig. 5A). The splenic artery and vein were ligated and divided using hemoclips and a vascular stapler, and the spleen was removed without complication (Fig. 5B).

**Figure 6.**
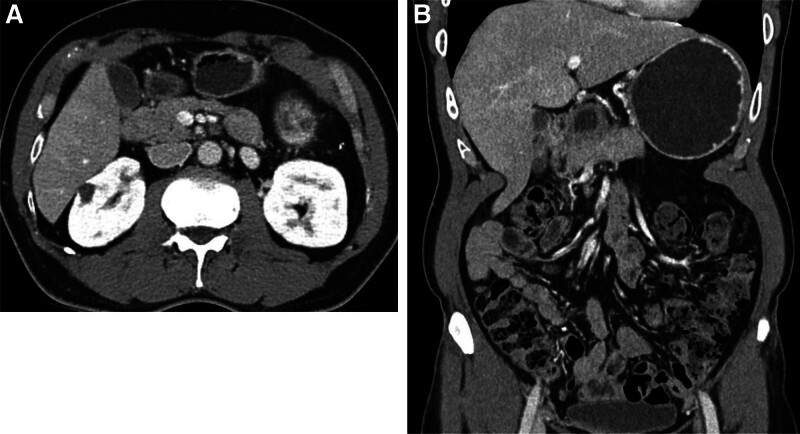
Postoperative follow-up CT. (A) Axial view of abdominal CT performed on May 10, 2024 (five months after surgery) showing resolution of previously engorged perigastric and splenic vein collaterals. (B) Coronal view confirming absence of abnormal varices or masses in the pelvic cavity 5 mo post-splenectomy. CT = computed tomography.

The postoperative course was uneventful, and the patient was discharged on postoperative day 3. A follow-up abdominal CT performed on May 10, 2024 (five months after surgery) revealed complete resolution of the perigastric varices and collateral veins, with no evidence of portal or splenic vein thrombus (Fig. 6). A concise clinical timeline summarizing the chronological sequence of key endoscopic, radiologic, and surgical findings is presented in Table 1.

**Table 1:** Clinical timeline of the patient.

Date/Hospital Day	Procedure/Event	Key Findings/ Outcome
2017	Screening EGD (other hospital)	IGV (type I), no bleeding stigmata
November 9, 2022	Surveillance EGD (our hospital)	IGV at fundus, no bleeding stigmata (Fig. 1A)
December 13, 2023 (HD1)	Admission/ Initial EGD	Large IGV type I without recent bleeding stigmata (Fig. 1B)
December 13, 2023 (HD1)	Abdominal CT	Wandering spleen in pelvic cavity; splenic vein engorgement; perigastric collaterals; no cirrhosis (Fig. 2)
December 13, 2023 (HD1)	Sigmoidoscopy	Fresh blood in rectosigmoid colon (Fig. 3A)
December 14, 2023 (HD2)	Colonoscopy	Normal colonic mucosa, no bleeding focus (Fig. 3B)
December 14, 2023 (HD2)	Capsule endoscopy	Normal jejunum; dark red fluid and yellowish debris in distal ileum, consistent with old blood (Fig. 3C and D)
December 20, 2023 (HD9)	EGD (Rebleeding episode)	Active spurting bleed from fundic varix → EVO with cyanoacrylate (Fig. 4A and B)
December 28, 2023 (HD17)	Laparoscopic splenectomy	Enlarged wandering spleen in pelvic cavity; congested perigastric varices; spleen removed successfully (Fig. 5A and B)
May 10, 2024	Follow-up CT	Complete regression of perigastric collaterals and splenic vein congestion, no thrombus (Fig. 6A and B)

CT = computed tomography, EGD = esophagogastroduodenoscopy, EVO = endoscopic variceal obturation, HD = hospital day.

## 3. Discussion

Wandering spleen is a rare clinical condition caused by the absence or laxity of the suspensory ligaments, such as the splenorenal and gastrosplenic ligaments, resulting in abnormal spleen mobility.^[9]^ This mobility predisposes the spleen to develop torsion of its vascular pedicle, which can compromise venous drainage and lead to complications such as infarction, hypersplenism, and vascular congestion.^[2]^ An uncommon variant of the splenic vein can lead to segmental (sinistral) portal hypertension and IGV.^[3]^ This condition is distinct from generalized portal hypertension in that it is not associated with liver disease, and esophageal varices are typically absent.

Although early imaging techniques such as contrast-enhanced CT, Doppler ultrasound, and magnetic resonance imaging are crucial for detecting splenic torsion and venous congestion,^[10]^ radiologic confirmation of gastric variceal regression after splenectomy has rarely been reported.^[3,8]^ For example, Tan et al (2007) and Singla et al (2008) described cases of wandering spleen with gastric variceal bleeding successfully managed with splenectomy, but radiologic follow-up was either absent or limited, and the regression of varices was inferred mainly from clinical improvement rather than objective imaging.^[4,7]^ The paucity of follow-up imaging data leaves a gap in understanding the dynamic reversibility of sinistral portal hypertension. Our case bridges this gap by providing radiological evidence of complete variceal resolution after definitive surgical correction.^[2,11]^

In our case, durable resolution of the varices was achieved only after laparoscopic splenectomy, as confirmed through a follow-up CT performed 5 months postoperatively showing complete regression of the perigastric collaterals and thrombi.^[12]^ Given that IGV were first identified in 2017 and the patient remained asymptomatic for several years before presenting with bleeding, it is plausible that the splenic torsion developed in a chronic or intermittent fashion. Such recurrent or partial twisting of the splenic pedicle may have caused gradual splenic venous congestion and progressive formation of gastric varices, eventually leading to hemorrhage rather than an acute torsion event typically associated with severe abdominal pain. This finding underscores the causal role of splenic venous outflow obstruction; however, long-term radiologic follow-up remains underreported in the literature. Although most published cases did not report recurrence once splenectomy was performed, our case emphasizes the unique contribution of imaging confirmation and the potential benefit of extended follow-up.

In adults, splenectomy is generally preferred over splenopexy, particularly when the spleen is enlarged, congested, or complicated by gastric variceal bleeding. Splenopexy is mainly reserved for children or young adults where splenic preservation is critical.^[13,14]^ Endoscopic cyanoacrylate injection provides only temporary hemostasis because it does not correct the underlying splenic vein outflow obstruction, which explains the risk of recurrent bleeding.^[15]^ Thus, splenectomy represents the definitive treatment for achieving durable hemodynamic correction.

Furthermore, this case emphasizes the diagnostic value of cross-sectional imaging modalities. Abdominal CT not only confirmed an ectopic spleen, but also visualized perigastric varices and splenic vein engorgement. The absence of cirrhotic features and esophageal varices supported the diagnosis of segmental portal hypertension. Although the patient had a history of substantial alcohol intake, laboratory tests and cross-sectional imaging definitively ruled out liver cirrhosis or portal hypertension of hepatic origin. This finding reinforces that the observed varices were attributable to isolated, sinistral portal hypertension secondary to splenic vein torsion rather than to alcohol-related liver disease.

In our patient, gastric varices were initially identified incidentally and remained stable for several years under conservative observation with periodic endoscopy and CT. Follow-up was later continued at a local screening center, where no interval change was noted. The subsequent bleeding episode likely reflected a chronic or intermittent torsion of the splenic vein. Although closer follow-up at a tertiary center might have prompted earlier surgical consideration, the patient’s asymptomatic course and lack of radiologic progression made conservative management appropriate until the onset of bleeding.

This case highlights the need for a heightened clinical suspicion of wandering spleen in patients presenting with IGV and no evidence of liver disease. When imaging identifies splenic displacement and venous congestion, timely surgical intervention controls bleeding and reverses the underlying hemodynamic changes. These insights support the application of splenectomy as a definitive, curative approach in select patients.

## 4. Conclusions

This case reinforces the need to consider wandering spleen as a potential cause of IGV in patients without liver disease. In such cases, splenic torsion may lead to sinistral portal hypertension and life-threatening bleeding which cannot be resolved with endoscopic therapy alone. Our findings demonstrate that splenectomy not only achieves durable hemostasis, but also enables regression of varices, underscoring the importance of timely imaging-based diagnosis and surgical intervention.

## Acknowledgments

The authors received no specific support, assistance, or donations in relation to this work. OpenAI’s ChatGPT was used to assist with manuscript structuring and refinement of clinical content. All content was reviewed and approved by the authors, and the manuscript was professionally edited prior to submission.

## Author contributions

**Conceptualization:** Byung Chul Jin, Seung Young Seo, Hyeon Seok Baek, Sang-Wook Kim.

**Supervision:** Seung Young Seo, Hyeon Seok Baek, Sang-Wook Kim.

**Visualization:** Byung Chul Jin.

**Writing – original draft:** Byung Chul Jin.

**Writing – review & editing:** Byung Chul Jin, Sang-Wook Kim.
